# Duplication digestive: à propos d´un cas néonatal

**DOI:** 10.11604/pamj.2021.38.353.28385

**Published:** 2021-04-12

**Authors:** Cyrine Belghith, Sawssam Armi, Souhir Najar, Nahla Ben Saada, Tahar Makhlouf, Nabil Mathlouthi, Olfa Slimani, Leila Attia

**Affiliations:** 1 Service de Gynécologie Obstétrique A, Hôpital Charles Nicolle, Tunis, Tunisie; 2 Faculté de Médecine de Tunis, Tunis, Tunisie

**Keywords:** Duplication digestive, diagnostic prénatal, rapport de cas, Gastrointestinal duplication, prenatal diagnosis, case report

## Abstract

Les duplications digestives sont définies comme des malformations tubulaires ou kystiques, siégeant sur un segment du tube digestif, de la cavité buccale à l´anus. Elles représentent une entité rare. Le diagnostic anténatal est possible quand il s´agit d´une forme kystique volumineuse. Nous rapportons le cas d´une patiente de 33 ans, primipare sans antécédents médicaux ou chirurgicaux dont la grossesse était mal suivie. Une seule échographie faite à 8 semaines d´aménorrhée (SA). Au cours du troisième trimestre l'examen échographique a montré une image kystique anéchogène de 3cm, à paroi propre, hautement situé dans le pelvis, non vascularisée. Cette image paraît de localisation antérieure, indépendante des reins et de la vessie. Afin de mieux explorer cette image et ses caractéristiques, une imagerie par résonance magnétique (IRM) fœtale a été réalisée montrant une image kystique à paroi propre, bien limitée, venant au contact des anses grêliques sur le versant mésentérique. Le diagnostic d´une duplication digestive a été fortement consolidé. L´accouchement s´est déroulé sans incidents par voie haute à 39 SA pour infertilité primaire de 7 ans. L´échographie post-natale a renforcé cette hypothèse, en montrant une formation kystique au niveau de l´hypochondre gauche, de 45 mm x 19 mm, multi cloisonnée, pouvant cadrer avec une duplication digestive. Le nouveau-né a été adressé à la consultation externe de chirurgie pédiatrique pour meilleure prise en charge et programmation d´un acte chirurgical dans les premiers 6 mois. La découverte d´une image fœtale kystique anéchogène pose le problème du diagnostic étiologique d´une part et du suivi et prise en charge post-natale d´autre part.

## Abstract (en)

Gastrointestinal duplications are defined as tubular or cystic malformations of a segment of the digestive tract, from the oral cavity to the anus. They are rare. Prenatal diagnosis ca be made in patients with a voluminous cyst. We here report the case of a 33-year-old primiparous woman with no previous medical-surgical history who was poorly screened. She just underwent ultrasound scan at 8 weeks of amenorrhea (WA). During the third trimester of pregnancy ultrasound showed anechoic nonvascularized cyst measuring 3cm located in the upper pelvis. It was anteriorly located; the kidneys and the bladder were not involved. Then magnetic resonance imaging (MRI) of the fetus was performed to better interpret ultrasound results. This showed well-defined cyst in contact with the small bowel loops along the mesenteric side. The diagnosis of gastrointestinal duplication was strongly suspected. C-section was done at 39 weeks’ gestation because the woman had a history of primary infertility (7 years). Delivery proceeded without complications. Postnatal ultrasound results reinforced the hypothesis of gastrointestinal duplication, showing a cystic mass with a multi-bulkhead-like structure at the level of the left hypochondrium, measuring 45 mm x 19 mm, which could be consistent with gastrointestinal duplication. The new-born was referred to the Paediatric Surgery for better management and surgery in the first 6 months of life. The discovery of fetal anechoic cyst poses a challenge in etiology and diagnosis on the one hand and of follow-up and postnatal management on the other hand.

## Introduction

Les progrès considérables réalisés en échographie ultrasonore au cours des dernières années permettent aujourd´hui de découvrir un certain nombre de malformations fœtales. La sensibilité du dépistage échographique des pathologies digestives progresse et donne l´opportunité de réaliser secondairement un bilan d´imagerie de diagnostic. Celui-ci permet de préciser le bilan lésionnel et de rechercher les malformations associées. Cette possibilité du diagnostic anténatal permet de mieux connaître la pathologie du tube digestif chez le fœtus et la mise en évidence des images kystiques intra-abdominales fœtales. Le diagnostic différentiel est important et comprend des anomalies du tractus gastro-intestinal, des voies urinaires, du système hépato-biliaire, de l'appareil reproducteur féminin, du pancréas, de la rate et du mésentère. L´anticipation apportée par le diagnostic prénatal permet une surveillance obstétricale adaptée et une organisation de la prise en charge néonatale dans une structure adaptée. Le présent article représente, au mieux de notre connaissance, une description de l'aspect prénatal d'un kyste de duplication iléale.

## Patient et observation

**Informations spécifiques au patient anonymisé, préoccupations et symptômes principaux du patient**.

**Antécédents médicaux, familiaux et psychosociaux, y compris les informations génétiques pertinentes**.

**Interventions passées pertinentes et leurs résultats**.

Il s´agissait d´une patiente âgée de 33 ans, sans antécédent pathologiques notables, primigeste, primipare. La grossesse actuelle était spontanée, après une Infertilité primaire de 7 ans, non explorée. C´était une grossesse mal suivie. En effet, l´échographie du 1^er^ et du 2^e^ trimestre étaient non faites. On ne disposait que d´une seule échographie faite à 8 SA. Les marqueurs sériques du 1^er^ et du 2^e^ trimestre étaient également non faits. La patiente s´était présentée en consultation devant la découverte à l´échographie du 3^e^ trimestre d´une image fœtale abdominale inexpliquée.

**Figure 1:**
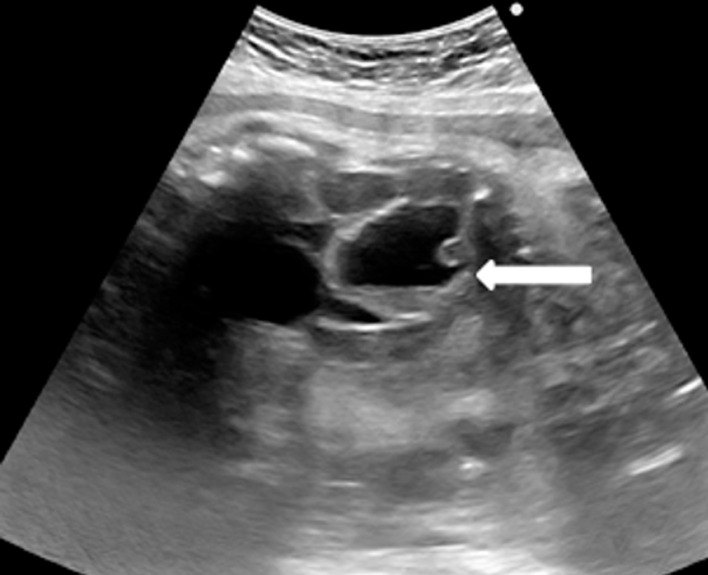
une image abdominale kystique anéchogène de 3 cm bien limitée latéralisée à gauche, à paroi propre, non vascularisée au doppler

**Résultats clinique:** l´examen clinique ne montrait pas d´anomalies. A l´échographie, faite à 32 SA, c´était une grossesse mono fœtale évolutive en présentation céphalique. Les biométries étaient en rapport avec le terme théorique. Un liquide amniotique en quantité normale et un placenta antéro-fundique normalement inséré. A l´étage abdominal, à son tiers moyen, on trouvait une image abdominale kystique anéchogène de 3 cm bien limitée latéralisée à gauche, à paroi propre, non vascularisée mobile, persistante tout au long de l´examen (Figure 1). Au cours de l´examen, on arrivait à bien visualiser une vessie tout indépendante de l´image. Les deux reins étaient en place, de taille et d´écho structure normale. L´estomac était également en place. Par ailleurs, pas d´autres anomalies morphologiques décelables. Le fœtus étant de sexe féminin.

**Figure 2:**
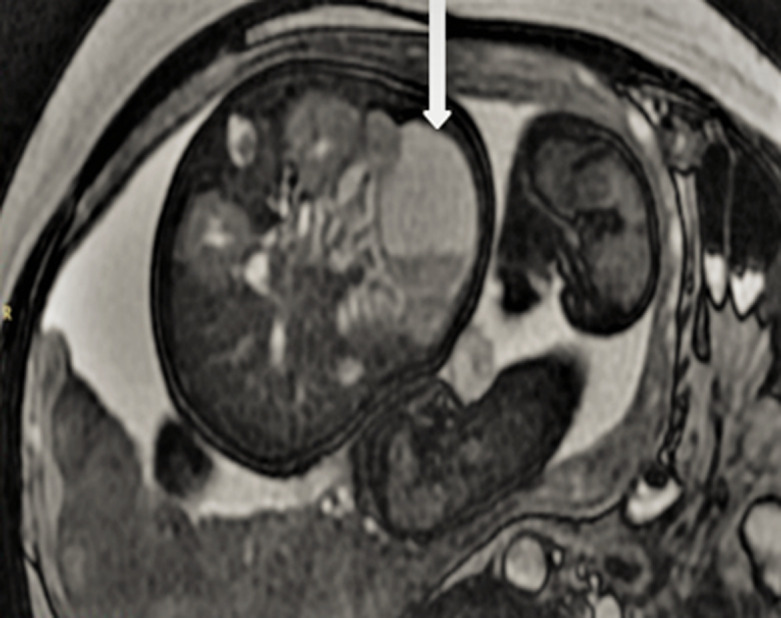
formation kystique, bien limitée à paroi propre en hyposignal T1 et hypersignal T2, venant au contact intime des anses digestives sur leur versant mésentérique

**Figure 3:**
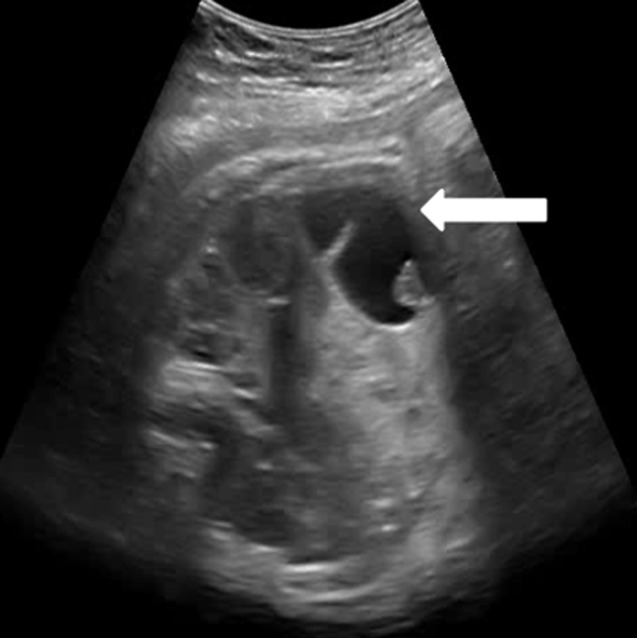
un aspect échographique stable de l´image intra-abdominale

**Chronologie, évaluation diagnostique:** devant ces constatations échographiques, trois diagnostics ont été évoqués: un kyste de l´ovaire, un lymphangiome kystique et une duplication digestive. Nous avons complété notre exploration par la réalisation d´une IRM fœtale qui a montré la présence d´une formation kystique, bien limitée à paroi propre en hyposignal T1 et hypersignal T2, venant au contact intime des anses digestives sur leur versant mésentérique (Figure 2). Les anses grêliques étaient non dilatées. Le foie, les voies biliaires, les reins et la vessie étaient intègres avec absence d´épanchement intra-abdominal et absence d´anomalie à l´étage cérébral. Le diagnostic de duplication digestive était évoqué en premier lieu. Un contrôle échographique a été réalisé à 35 SA et 37 SA montrant un aspect échographique stable de l´image intra-abdominale (Figure 3).

**Figure 4:**
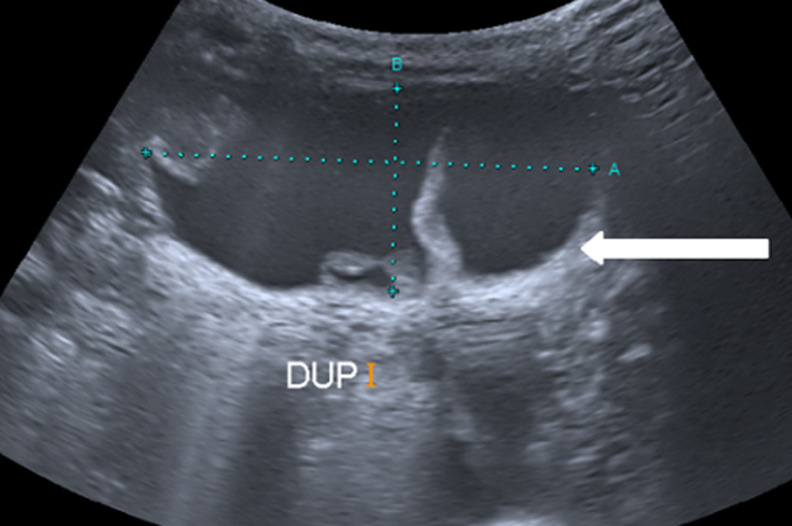
formation kystique au niveau de l´hypochondre gauche, de 45 mm x 19 mm, multi cloisonnée, pouvant cadrer avec une duplication digestive iléale non compliquée

**Intervention thérapeutique:** l´accouchement s´est déroulé sans incidents par césarienne à 39 SA pour infertilité primaire de 7 ans. Le nouveau-né était de sexe féminin avec un poids de naissance à 3200g, Apgar 9-10. L´examen à la naissance était sans anomalie, notamment les choanes, l´œsophage et l´anus étaient perméables. Le bébé a été mis sous-alimentation orale avec surveillance rapprochée et on n´a pas constaté de syndrome occlusif. Au bilan néonatal, une radio-thorax faite à la naissance avait montré une sonde gastrique en place au niveau de l´abdomen, un tube digestif aéré et une silhouette cardiaque d´aspect normale. Une échographie abdominale réalisée immédiatement après la naissance concluait à un foie de taille normale, de contours réguliers et d´écho structure homogène sans lésion focale décelable. Le tronc porte et les veines sus hépatiques étaient perméables. La vésicule biliaire était à paroi fine, non lithiasique. Les deux reins étaient de taille normale de contours réguliers avec une bonne différenciation cortico-médullaire. Absence d´adénomégalie intra-abdominale et d´épanchement intra péritonéal Présence d´une formation kystique au niveau de l´hypochondre gauche, de 45 mm x 19 mm, multi cloisonnée, pouvant cadrer avec une duplication digestive iléale non compliquée (Figure 4).

**Suivi et résultats:** devant cette duplication digestive asymptomatique, le nouveau-né a été adressé à la consultation externe de chirurgie pédiatrique pour meilleure prise en charge et programmation d´un acte chirurgical dans les 6 premiers mois. Les parents ont été informés des signes cliniques d´une éventuelle complication.

## Discussion

Les duplications digestives sont des anomalies du développement intestinal par anomalie de reperméabilisation du tractus digestif et se produisent dans environ 1 sur 4500 naissances [1]. Ils peuvent survenir à tout point le long du tractus gastro-intestinal et sont le plus souvent diagnostiquées en anténatal sous la forme de lésions kystiques abdominales [2, 3]. Bien que les duplications du tube digestif soient rares, leur localisation grêlique est de loin la plus fréquente, avec une nette prédominance pour les dernières anses iléales [4]. En effet, la duplication de la grêle représente 55 à 60% des duplications digestives et touche plus souvent l´iléon que le jéjunum. Les autres localisations classiques sont par ordre de fréquence décroissant: l´œsophage, le côlon, le jéjunum, l´estomac et le duodénum. Si la définition anatomopathologique de la duplication est unanimement admise, sa pathogénie reste très discutée. Plusieurs théories ont été avancées; la théorie vasculaire: une anomalie de différenciation embryonnaire sans qu´aucune ne puisse expliquer le polymorphisme topographique, l´association à d´autres malformations et l´existence d´hétérotopie gastrique ou pancréatique. Des anomalies associées peuvent survenir jusqu´à 50% des cas et peuvent inclure d'autres duplications du tube digestif et des anomalies vertébrales [5]. Les critères recommandés pour le diagnostic de la duplication digestive comprennent: une image kystique à la proximité du tube digestif, une vascularisation commune avec le tube digestif et l´étendue de la couche musculaire lisse externe [6].

Ainsi, les duplications correspondent à une malformation se produisant au cours de la phase embryonnaire. En conséquence, elles peuvent-être identifiées lors de la première échographie à 12 semaines d´aménorrhées sous la forme d´un kyste, bien qu´en pratique la découverte de l´image kystique soit plus classique au deuxième trimestre. Pour notre patiente, le diagnostic était tardif car la grossesse était mal suivie. L´aspect échographique typique est celui d´une structure anéchogène, à paroi épaisse, faite d´une couche interne hyperéchogène et d´une couche périphérique hypoéchogène. Le second élément important du diagnostic est le contact intime avec une structure digestive ce qui est habituellement facile à voir dans la région iléo-cæcale. Ailleurs, la mobilité de la lésion est parallèle au péristaltisme digestif. On peut également observer une variabilité de l´aspect échographique au cours de la surveillance [7]. Le diagnostic différentiel, habituellement facile, se fait avec le kyste de l´ovaire, le lymphangiome kystique et le kyste du cholédoque qui est plus postérieur et dont la paroi est plus fine. L'aspect tubulaire, démontrant des mouvements péristaltiques et les signes d'obstruction intestinale à l'échographie prénatale et à l'imagerie par résonance magnétique fœtale, peuvent aider à différencier les duplications entériques des autres structures kystiques thoraco-abdominales. Enfin, ces duplications digestives sont généralement uniques mais peuvent être multiples. L´association d´une duplication œsophagienne à une duplication grêlique est la plus classique [8].

Le diagnostic néonatal est fréquent d´autant que celui-ci est souvent évoqué en période fœtale. Le caractère pathologique de la duplication digestive réside essentiellement dans les complications potentielles qui peuvent être prévenues par la réalisation d´une chirurgie précoce. Les complications possibles sont les occlusions, les dilatations majeures de l´anse dupliquée, et les invaginations qui mènent alors à une chirurgie urgente. La mise au point néonatale se limitera à la confirmation échographique du diagnostic anténatal et au suivi clinique jusqu´à la chirurgie qui devra être pratiquée (hormis toute complication) au cours de la première année de vie.

## Conclusion

Devant une image kystique intra-abdominale, l´échographie est l´examen de première intention. L´utilisation d´une sonde haute fréquence facilite la mise en évidence d´une paroi stratifiée qui permet d´évoquer le diagnostic de duplication digestive qui reste accessible au diagnostic anténatal surtout au 2^e^ trimestre. L´introduction de l´IRM digestive fœtale permet maintenant un bilan malformatif précis et une nette amélioration de l´orientation étiologique. Le diagnostic anténatal aide ainsi à préparer la famille et les équipes médicales à une prise en charge rapide et ciblée. Cette pathologie étant rare, de bon pronostic, et son traitement est toujours chirurgicale.

## References

[1] Puligandla PS, Nguyen LT, St-Vil D, Flageole H, Bensoussan AL, Nguyen VH (2003). Gastrointestinal duplications. J Pediatr Surg.

[2] Thompson SK, Wong AL, Trevenen CL, MacGregor JH (2004). Enteric duplication cyst. Am J Surg.

[3] Ahmet Gul, Gungor Tekoglu, Halil Aslan, Altan Cebeci, Onur Erol, Murat Unal (2004). Prenatal sonographic features of eosophageal and ileal duplications at 18 weeks of gestation. Prenat diagn.

[4] Corteville JE, Gray DL, Langer JC (1996). Bowel abnormalities in the fetuscorrelation of prenatal ultrasonographic findings with outcome. Am J Obstet Gynecol.

[5] Dhawal Sharma, Bharany RP, Mapshekhar RV (2013). Duplication cyst of pyloric canal: a rare cause of pediatric gastric outlet obstruction (rare case report). Indian J Surg.

[6] Veyrac C, Couture A, Saguintaah M, Baud C (2004). MRI of fetal GI tract abnormalities. Abdom Imaging.

[7] Chen M, Lam YH, Lin CL, Chan KW, Hui PW, Hoi Yin (2002). Sonographic features of ileal duplication cyst at 12 weeks. Prenat Diagn.

[8] Noël L, Becmeur F, Jacques C, Langer B, Marcellin L, Dietemann J (2001). Duplication digestive multiple: à propos d´un cas néonatal. J Radiol.

